# Dignity of Older Adults in Long-Term Care Facilities: A Systematic Review of Qualitative Evidence from Residents, Staff, and Relatives

**DOI:** 10.3390/healthcare13222839

**Published:** 2025-11-08

**Authors:** Dong-Mei Xue, Dan-Ni Wang, Ying Bian

**Affiliations:** 1 State Key Laboratory of Mechanism and Quality of Chinese Medicine, University of Macau, Macao Special Administrative Region 999078, China; yc37530@um.edu.mo (D.-M.X.); mc15337@connect.um.edu.mo (D.-N.W.); 2 Institute of Chinese Medical Sciences, University of Macau, Macao Special Administrative Region 999078, China; 3 Department of Public Health and Medicinal Administration, Faculty of Health Sciences, University of Macau, Macao Special Administrative Region 999078, China

**Keywords:** dignity, older adult, long-term care facility, qualitative evidence

## Abstract

Background/Objectives: With global population aging, long-term care (LTC) facilities play an essential role in supporting older adults. However, residents are vulnerable to dignity loss in these institutional settings. Understanding dignity from the perspectives of residents, staff, and relatives is critical for informing person-centered care and policy. This review aimed to synthesize qualitative evidence on dignity in LTC facilities across multiple stakeholders. Methods: Following the PRISMA guidelines, we systematically searched six databases (PubMed, Scopus, Web of Science, Wan Fang, VIP, and CNKI) for qualitative studies published in 2010–2023. Eligible studies explored experiences of dignity among older LTC residents, staff, or relatives. Methodological quality was appraised using the Critical Appraisal Skills Programme (CASP) checklist. Data were analysed through thematic synthesis, and findings were compared across stakeholder groups. Results: A total of 1948 records were identified, of which 27 qualitative studies met the inclusion criteria. Two themes emerged from residents’ perspectives: institutionalization and resilience in preserving dignity. From staff perspectives, three themes were identified: understaffing and workload pressures, general approaches to dignity care, and person-centered care. Relatives’ accounts highlighted two themes: uneasiness regarding indignity and ethical expectations in a caring culture. Integrating these perspectives, we propose a triangular model in which residents, staff, and relatives collaboratively sustain dignity. Conclusions: Older adults’ dignity in LTC is shaped by complex interactions among institutional conditions, caregiving practices, and family involvement. Enhancing dignity requires adequate staffing, structural support for person-centered care, and greater involvement of relatives in decision-making. The proposed triangular model offers a framework for guiding interventions, staff training, and policy reforms aimed at safeguarding dignity in LTC facilities.

## 1. Introduction

Ageing is a universal challenge for humanity. The proportion of people aged over 60 is projected to nearly double between 2015 and 2050, increasing from 12% to 22% worldwide [1]. As populations grow older, the demand for long-term care (LTC) facilities inevitably rises. Safeguarding the dignity of older adults living in LTC facilities has therefore become a central focus of national elderly care policies [2,3,4,5,6,7]. The UN Decade of Healthy Ageing (2021–2030) calls for multisectoral collaboration to ensure healthy ageing worldwide [8]. The European Union’s 2022 Council Recommendation promotes affordable, high-quality LTC and stronger protection for formal and informal carers [9]. Similarly, China’s 14th Five-Year Plan (2021–2025) [10], Australia’s 2024 Aged Care Act [11], and the United States’ 2024 National Plan on Aging [12] all emphasise equitable access, quality assurance, and human rights in aged care. Within these global efforts, preserving the dignity of older adults stands as a central ethical and practical concern, yet evidence shows that residents in LTC facilities remain at heightened risk of dignity loss [13].

The concept of dignity originates from the Latin *dignitas*. Within the classical Kantian tradition, dignity is regarded as an absolute value that cannot be replaced, weighted, or traded [14]. Edlund et al. have distinguished between two dimensions of dignity: “human dignity” and “social dignity” [15]. Human dignity is innate and absolute, emphasising inner freedom that cannot be taken away, measured, or destroyed [16]. In contrast, social dignity—also referred to as personal dignity—is a subjective construct shaped by social relationships [16,17]. As it can be promoted or undermined by the care environment, social dignity is of particular importance in the processes of illness and ageing [16].

Building on this distinction, Nordenfelt has advanced a four-dimensional framework of dignity in older people: human dignity (inherent in all individuals), dignity of identity (derived from physical and mental integrity), dignity as moral stature (linked to self-esteem, character, and virtue), and dignity of merit (influenced by social status and connections) [18]. Among these, the dignity of identity is often regarded as the most crucial in later life, especially in institutional care settings where both physical and psychological vulnerability are heightened. Accordingly, this study places particular emphasis on the physical and mental integrity of older adults in LTC facilities.

Several reviews have explored the dignity of older adults in LTC facilities (see Table 1) [13,19,20]. However, all were published before 2020 and were mainly concept- or narrative-based rather than systematic. Most included only English studies conducted in Europe and North America. Research from Asian regions, particularly from China, remains scarce in the existing literature. To address these gaps, this review systematically identifies, appraises, and synthesises qualitative evidence on the dignity of older adults in LTC facilities from the perspectives of residents, staff, and relatives. By comparing dignity experiences across different roles and regional contexts, the findings aim to inform practice and policy improvements LTC systems.

## 2. Materials and Methods

**Figure 1:**
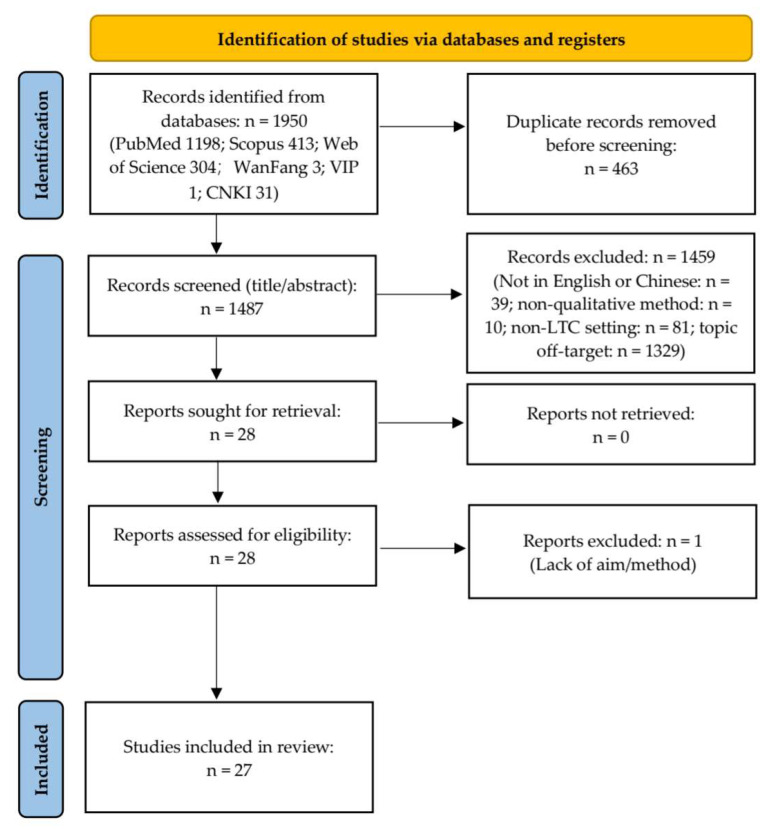
PRISMA 2020 flow diagram of the study selection process. A total of 1950 records were identified through database searching and additional sources, with 27 studies finally included in the qualitative synthesis.

This review was conducted in accordance with the Preferred Reporting Items for Systematic Reviews and Meta-Analyses (PRISMA 2020) guidelines (see Figure 1 and Appendix A Table A5). At the initial stage of this project, we were not fully aware of the requirement for registration, thus no prospective protocol was registered before data analysis. The review has been retrospectively registered in the Open Science Framework (OSF): https://osf.io/kn5sa (accessed on 30 September 2025). OSF was chosen over PROSPERO because the latter mainly provides registration forms for intervention-based reviews and does not allow retrospective registration [21]. Given that this review synthesised qualitative evidence without interventions, OSF offered a more appropriate and flexible platform under the category “Registration following analysis of the data”. As the review analysed only previously published studies and did not involve human participants or animals, ethical approval was not required.

### 2.1. PICo Framework for Qualitative Systematic Review

Consistent with the Joanna Briggs Institute (JBI) methodological guidance for qualitative systematic reviews, the review question and inclusion criteria were structured using the PICo (Population, Phenomena of Interest, Context) framework rather than the traditional PICO (Population, Intervention, Comparison, Outcome) format [22]. PICO is primarily designed for reviews of effectiveness that compare interventions and outcomes, whereas qualitative systematic reviews aim to explore meanings, experiences, and contextual factors underlying human phenomena. Therefore, “a different approach is required” for qualitative synthesis, and PICo provides an appropriate structure for framing research questions and defining eligibility criteria in this context [22]. This review follows the PICo framework to synthesise qualitative evidence on the dignity of older adults in LTC facilities. Specifically, it seeks to integrate perspectives from residents, staff, and relatives, and to identify both the commonalities and the differences in how these groups perceive and experience dignity (See Table 2).

### 2.2. Eligibility Criteria

Studies were eligible for inclusion if they adopted a qualitative design and explicitly explored the dignity of older adults (aged 60 years or above) living in LTC facilities, as reported from the perspectives of residents themselves, professional caregivers, or relatives. Given that this review focuses on the current state of dignity among older adults in LTC facilities rather than its historical evolution, and considering that these experiences are closely linked to contemporary ageing and LTC policy contexts, we restricted the review to studies published from 2010 to 2023. Considering that the research team works primarily in English and Chinese, only articles written in these two languages were included, retrieved from three English (PubMed, Scopus, Web of Science) and three Chinese (Wan Fang, VIP, CNKI) databases. This decision was made to ensure accurate interpretation and reliable synthesis of qualitative evidence. Because dignity is an inherently qualitative concept, we focused exclusively on qualitative studies to capture subjective experiences in depth. Exclusion criteria were quantitative, mixed-methods, systematic review or meta-analytic studies, and research conducted outside institutional LTC contexts (e.g., home care or hospital care).

### 2.3. Search Strategies

A systematic search was conducted in six electronic databases: PubMed, Scopus, Web of Science, Wan Fang, VIP, and CNKI. Searches were limited to the title and abstract (or title/abstract/keywords in Chinese databases) to ensure specificity. The time frame was set from 1 January 2010 to 31 December 2023. Both controlled vocabulary and free-text terms were used, covering four domains: older adults, dignity, qualitative research, and LTC facilities. The search strategy was iteratively refined through team discussion to balance comprehensiveness and feasibility given team language capacity. The full list of search terms is summarized in Table 3, and sample search strategies for PubMed and Wan Fang are provided in Appendix A Table A1.

### 2.4. Study Selection and Quality Appraisal

All retrieved records were imported into EndNote, and duplicates were removed. Two reviewers independently screened titles, abstracts and full texts against eligibility criteria. Discrepancies were resolved through discussion or consultation with a third reviewer. The methodological quality of the included studies was assessed using the Critical Appraisal Skills Programme (CASP) Qualitative Checklist 2018 (See Appendix A Table A2). The checklist consists of ten items assessing study validity, design, sampling, data collection, reflexivity, ethical issues, analysis, findings, and overall value. Each item was scored on a three-point scale (1–3), with total scores ranging from 8 to 24 [23]. Rather than excluding low-scoring studies, appraisal outcomes informed interpretation and weighting of findings.

### 2.5. Data Extraction and Synthesis

Data extraction included author, year, country/region, aim, methodology, participants and main findings, following the principles of thematic synthesis [24]. All data were imported into NVivo 12 for analysis in three stages: (1) line-by-line coding of findings, (2) development of descriptive themes, and (3) generation of analytical themes. The research team comprised three members with backgrounds in medicinal administration, holding undergraduate degrees in clinical medicine, nursing and health statistics. The first and second authors independently conducted coding and theme development before joint discussion. Monthly team meetings were held to review and refine themes. Where disagreement persisted after discussion, the third author adjudicated. This iterative process ensured depth and authenticity while maintaining reflexivity regarding potential disciplinary biases.

## 3. Results

The database search yielded 1950 records. In accordance with the PRISMA 2020 guidelines, 463 duplicates were removed, leaving 1487 records for title and abstract screening. From these, 28 articles were retrieved for full-text assessment, and one was subsequently excluded owing to the absence of a clear aim and methodological description. Ultimately, 27 qualitative studies were included in the final synthesis (See Figure 1). Methodological quality was appraised using the CASP Qualitative Checklist, with nine studies achieving the maximum score of 24 points (Appendix A Table A3). A descriptive summary of the included studies is provided in Appendix A Table A4. Thematic synthesis revealed perspectives on the dignity of older adults in LTC facilities across three stakeholder groups: residents, caregivers, and relatives. The key themes and subthemes identified within each perspective are summarised in Table 4.

**Figure 2:**
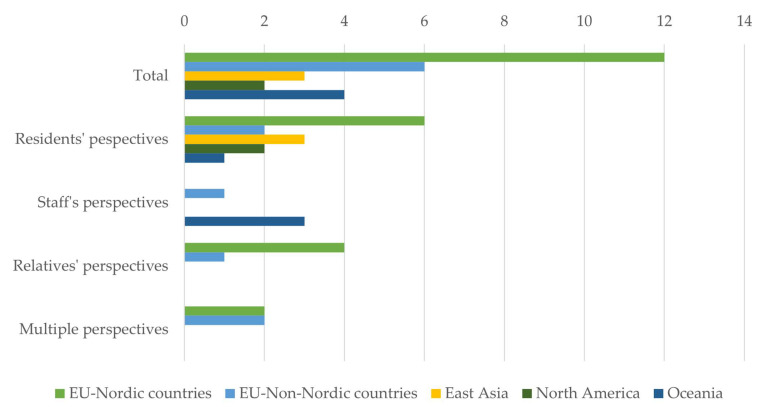
Geographical distribution of included studies.

The 27 included studies covered five major regions (See Figure 2): EU-Nordic countries (*n* = 12), EU-non-Nordic countries (*n* = 6), Oceania (*n* = 4), East Asia (*n* = 3), and North America (*n* = 2). From a perspective-based comparison, studies focusing on older adults were most common across all regions (*n* = 14), including EU-Nordic countries (*n* = 6), EU-non-Nordic countries (*n* = 2), East Asia (*n* = 3), and North America (*n* = 2). Research from staff perspectives was concentrated in Oceania (*n* = 3) and EU-non-Nordic countries (*n* = 1). Studies exploring relatives’ perspectives were primarily found in EU-Nordic (*n* = 4) and EU-non-Nordic regions (*n* = 1). Similarly, multiple-perspective studies were identified only in European countries, including EU-Nordic (*n* = 2) and EU-non-Nordic (*n* = 2).

### 3.1. Older Residents’ Perspectives

#### 3.1.1. Institutionalisation and Loss of Autonomy

Older people in LTC frequently expressed negative attitudes towards institutionalisation, which they perceived as eroding their dignity, independence, and autonomy [25]. Within this theme, four interrelated subthemes were identified: rigid routines and staff workload limit autonomy, “home” versus “facility”: privacy, freedom, and restriction, person-centred care: gap between claim and practice, and frailty and substituted decision-making.

##### Rigid Routines and Staff Workload Limit Autonomy

Heavy staff workload combined with rigid institutional routines was repeatedly associated with loss of dignity. Daily tasks in nursing homes were described as monotonous and time-consuming, leaving little capacity to address residents’ individual needs. The “endless wait” was often reported, whereby residents requiring assistance—sometimes even in urgent circumstances—were left unattended due to staff time constraints [26]. Scheduled routines also curtailed residents’ autonomy, as care was imposed according to institutional timetables rather than personal preferences [27]. Bathing and toileting, for instance, were typically allocated fixed times irrespective of residents’ wishes.

A key factor underlying these constraints was high staff turnover. Donnelly et al. [27] noted that residents were rarely cared for by the same staff member consistently. This lack of continuity was perceived as undermining trust and stability, whereas permanent staff were seen as more capable of preserving residents’ dignity [25]. Frequent turnover, particularly among nurses, left residents feeling isolated and insecure, given that these caregivers often represented their most reliable source of support [28].

##### “Home” Versus “Facility”: Privacy, Freedom, and Restriction

Although LTC facilities often aim to create a home-like environment, older adults frequently highlight the stark contrast between the notions of “home” and “facility”. For them, “home” symbolises privacy, autonomy, and freedom, whereas “facility” evokes exposure and restriction [29]. Privacy is threatened when individuals are required to share rooms [4], with their sense of dignity depending heavily on their relationship with roommates [30]. Non-consensual exposure, such as being seen naked by other residents or staff of the opposite sex, was also described as humiliating and harmful to dignity [31]. Beyond privacy, the concept of “home” conveys belonging, accumulated memories, and habitual routines, which are difficult to replicate within institutional environments [26,29]. Nonetheless, dignity can be supported when older adults are offered private rooms and permitted to keep personal items that connect them to their former homes [4].

Another crucial aspect of dignity is the right to freedom [3]. Moving from home to an LTC facility was often experienced as a sudden loss of liberty, especially regarding freedom of movement and expression. Restrictions on leaving the facility left some older people sitting idle for entire days, or even confined to their rooms, resulting in boredom and a diminished sense of dignity [25,26,32].Freedom of speech was also repeatedly stressed as a concern. Many older adults felt reluctant to voice complaints, fearing retaliation or feelings of guilt in their children [3,4,27,28]. Moreover, seeking assistance from staff was sometimes perceived as undermining autonomy, as reliance on others could be equated with loss of dignity. Ironically, asking for help should ideally serve as a way of preserving dignity. Whether help enhances or undermines dignity largely depends on how it is delivered by caregivers [2].

##### Person-Centred Care: Gap Between Claim and Practice

Although many LTC facilities claim to provide person-centred care, older adults often regard it as a slogan lacking real substance. This perception is reflected in three areas: diminished autonomy in daily activities, unsatisfactory staff attitudes, and shortcomings in environmental design.

Activities that are intended to preserve dignity should be tailored to individual preferences so that older people can participate actively [32]. Høy et al. [2] emphasised that personal initiative, rather than the activity itself, is the key to dignity-enhancing engagement. Older adults also need the right to decline participation and to decide which activities they wish to pursue [3].

The quality of person-centred care is closely tied to staff commitment and empathy. Caregivers who are dedicated and compassionate are more likely to create a sense of comfort and respect, whereas task-focused staff risk leaving a negative impression and even undermining dignity [27]. A lack of empathy can also lead to objectification, for example, when nurses reduce individuals with dementia to their diagnosis and focus solely on cognitive deficits. Such objectification was described as directly harmful to dignity [29].

Several studies also highlighted environmental limitations that hinder person-centred care. Design flaws, such as inadequate bedding, and the absence of personalised physical spaces were noted as barriers [33]. LTC facilities are therefore expected to provide environments that accommodate personal preferences and health conditions, thereby supporting the preservation of dignity [28].

##### Frailty and Substituted Decision-Making

Older adults in LTC facilities often experience losses of their “bodily selves”, such as impairments in hearing, vision, speech, or mobility. These physical limitations in themselves do not necessarily compromise dignity; rather, dignity is affected by how others respond to such vulnerabilities [2]. When compassionate care is provided, many older people with reduced mobility continue to feel respected. In contrast, neglect or objectification due to frailty was described as profoundly damaging to dignity. Physical rehabilitation or support that enables individuals to regain some bodily control can strengthen dignity, whereas dependence on intimate personal care because of severe illness was often perceived as diminishing it [30].

Frailty was also linked to the proxying of decisions. In many cases, the decision to enter an LTC facility, as well as day-to-day arrangements, was made jointly by family members and staff, leaving older people with little or no say in the process [28]. Yet older adults consistently expressed a desire to participate in decisions about their health and to remain part of the network of discussions regarding their care [33].

#### 3.1.2. Resilience and Adaptation to Preserve Dignity

Although many older adults expressed negative views of institutionalisation, they often demonstrated perseverance and sought to adapt in order to maintain their dignity [27]. This theme encompasses two subthemes: mourning past life and identity changes and finding benefits and cherishing small blessings.

##### Mourning Past Life and Identity Changes

Moving from home to an LTC facility frequently brought profound disruptions to social identity and relationships, regardless of whether the move was voluntary [26]. In their previous lives, older people enjoyed freedom to choose activities, organise their routines, and sustain close ties with family and friends [30]. Admission to an LTC facility disrupted these connections, severing them from former activities and long-established social networks [28]. Many newly admitted individuals expressed hopes of returning home or at least receiving regular visits from relatives, yet such visits often declined or ceased altogether [25,26,28,30]. This situation gave rise to strong negative emotions, including loneliness, insecurity from having no one to confide in, feelings of abandonment by children, and a perception of being a burden to both family and society [26,28].

Over time, older adults came to acknowledge that the “good old days” were gone. Nevertheless, their past experiences continued to shape their sense of self, creating continuity in personal identity. The past was used as a lens through which to interpret the present, evoking both grief for what had been lost and occasional positive sentiments [4].

##### Finding Benefits and Cherishing Small Blessings

To sustain dignity, older adults often adopted a positive attitude by focusing on the present. Some perceived LTC facilities as safer than their previous homes, while others valued no longer having to worry about being a burden on their families [4,28]. Being surrounded by peers in similar circumstances was also described as protective against disrespect from the wider social environment [30].

In adapting to change, many sought to maintain habits and routines from their past lives, thereby preserving continuity in self-identity [26]. At the same time, older people participated in social and cultural activities provided by LTC facilities, especially those they were already familiar with, and felt respected when their contributions were appreciated [4]. Some attempted to build supportive networks within the facility, for example, by developing relationships with nurses and fellow residents, to foster a sense of belonging [26,30]. Although new friendships were anticipated, only a few were successfully formed. To cope with this limitation, older adults often recalled or even idealised memories of the past as a means of remaining optimistic [2,25,28].

Attention to personal appearance was another way of maintaining dignity. Even when there was no need to go outside, some older adults reported taking care to remain neat and tidy as an expression of self-respect. Cherishing small blessings also played an important role: family visits were valued, and simple gestures of kindness from staff were described as deeply meaningful [4].

Overall, adaptation to life in LTC facilities was portrayed as a process of enduring threats and losses while striving to preserve dignity. This process was closely tied to identity, with successful adaptation fostering reciprocal and respectful relationships between older adults, fellow residents, and staff [2,33].

### 3.2. Staff’s Perspective

#### 3.2.1. Foundations of Dignified Care

Delivering dignified and personalised care requires staff to uphold professional standards in everyday practice and to maintain constructive partnerships with relatives.

##### Professional Standards in Everyday Care

Professionalism was described as the ability of staff to perform routine tasks competently while preserving the dignity of older adults. “Information gathering” before admission—covering religion, spirituality, habits, and personal preferences—was identified as an important step in understanding an individual’s background [34,35]. However, such information was often undervalued and only consulted when it directly influenced other tasks. Staff also carried the responsibility of ensuring cleanliness, privacy, and respect in daily care, particularly in situations where dignity could easily be compromised, such as incontinence [36]. Timely and sensitive responses in these moments were viewed as essential to alleviating embarrassment and reinforcing a sense of respect. Professionalism in this context therefore went beyond technical competence, requiring attentiveness, discretion, and a commitment to safeguarding dignity in routine interactions.

##### Partnership with Relatives

Effective communication with relatives was also considered integral to dignified care. Staff were regarded as the bridge between families and older people, facilitating emotional connection as well as continuity of care. Relatives provided valuable information about medical history, cognitive status, and personal preferences, which enabled staff to anticipate situations that might distress or demean older adults [34,36,37]. Such collaboration was seen not only as a way of protecting dignity but also as a means of building trust between families and care providers. By involving relatives in care planning and decision-making, staff could enhance older people’s well-being and strengthen the overall culture of respect within LTC facilities.

#### 3.2.2. Implementing Person-Centred Care

The social dignity of older adults is highly vulnerable to the care environment [16]. To safeguard dignity, staff must be attentive and empathetic, treating individuals as they themselves would wish to be treated. Two subthemes were identified: attentiveness to individual details and empathetic reciprocity in care.

##### Attentiveness to Individual Details

Staff who were observant of small details were better able to recognise and support the dignity of older people, working in partnership with them to achieve reciprocity. The experience of dignity varied by personality, cognitive ability, and resilience to stress, meaning that staff often adjusted their approach according to individual adaptability [35]. Examples included handling potentially embarrassing situations with sensitivity and engaging in casual conversation to ease discomfort. Attentive staff also recognised the importance of faith and spirituality in preserving dignity [34].

##### Empathetic Reciprocity in Care

Many caregivers reported drawing upon their own preferences to guide their care practices, adopting the principle of “treating others as you would like to be treated” [35]. This approach was aimed at protecting the subjective and personal dignity of older adults and shielding them from the judgement of others [36]. Different professional groups emphasised distinct aspects of dignity: physicians tended to prioritise physical functioning and independence (e.g., providing electric wheelchairs), while nurses focused on the quality of daily care, such as bathing preferences. Staff who were particularly conscious of privacy stressed environmental privacy (e.g., knocking before entering a room), physical privacy (ensuring older adults were not exposed when undressed), and confidentiality (avoiding discussions of health conditions in the presence of others) [35]. Overall, protecting dignity in these ways was described as mutually rewarding, fostering a reciprocal sense of respect and satisfaction between staff and those receiving care.

#### 3.2.3. Workforce Shortages and Workload Pressures

Consistent with the views of older adults, staff in LTC facilities often attributed threats to dignity to insufficient personnel [30,35]. This shortage was seen as both a symptom and a cause of demoralisation within the aged-care sector. Two subthemes were identified: time pressure and paperwork burden and high turnover and low morale.

##### Time Pressure and Paperwork Burden

Although staff considered the provision of person-centred care fundamental to preserving dignity, they acknowledged the difficulty of achieving this in practice [35]. Ideally, care should be tailored to the health conditions, interests, and preferences of older people, while building trusting relationships. However, such goals were difficult to realise under strict time constraints. Dignity violations were most likely when staffing shortages forced workers to follow rigid routines, complete tasks hastily, and devote insufficient attention to individuals. The heavy administrative workload further reduced the time available for meaningful interactions, diverting staff energy away from direct care [34,35,36].

##### High Turnover and Low Morale

High staff turnover, low morale, and the undervaluation of the aged-care profession were repeatedly described as barriers to sustaining dignified care. Many studies referred to the heavy and relentless workload and noted that institutionalisation compounded these challenges [35]. Staff also perceived the social status of aged-care practitioners as low, contributing to professional frustration and attrition. Importantly, participants emphasised that protecting the dignity of older adults required simultaneous attention to the occupational dignity of caregivers themselves. Improving working conditions—such as by streamlining routine tasks, reducing paperwork, and ensuring adequate staffing and remuneration—was considered essential for both retaining staff and enabling them to provide respectful and dignified care [34,35].

### 3.3. Relatives’ Perspective

Relatives play multiple roles in LTC facilities: they are visitors, observers, and occasional participants in daily life. As family caregivers, they remain deeply concerned about their loved ones and closely observe changes in their dignity. From the analysis, two themes were identified: unease regarding neglect and indignity and expectations for an ethical, caring culture.

#### 3.3.1. Unease Regarding Neglect and Indignity

Many studies reported that when older adults were first admitted to nursing homes, relatives initially felt reassured, believing they had secured a safe environment where quality care would be provided. However, over time, they often became uneasy as they observed instances where staff failed to meet the needs of older people or even delivered care perceived as humiliating or degrading [5,6].

##### Perceived Lack of Empathetic Care

Relatives consistently stressed that dignity is an inalienable human right grounded in individuality and integrity [5]. Acting as both observers and participants, they often interpreted the experiences of older adults as if these were happening to themselves. This dual perspective exposed them to a sense of vulnerability and dependency, amplifying both the promotion and the violation of dignity.

While staff frequently claimed to follow the principle of “treating others as you would like to be treated”, relatives felt this was rarely practised in reality. Instead, they perceived care as contributing to abandonment, manifesting as emptiness, helplessness, loneliness, and insecurity [6,7]. Relatives distinguished between “concrete abandonment”—such as being left unattended in rooms or toilets, or segregated due to cognitive impairment—and “existential abandonment”, in which needs were unnoticed or dismissed, such as enduring the “endless wait”, being infantilised, or being belittled because of disability [7]. Staff indifference, or the tendency to objectify older adults, was seen as intensifying these experiences. Relatives reported sharing the distress of their loved ones, resulting in a strong sense of unease [5,6,38].

##### Reluctant Reliance on the System

Relatives often expressed conflicting attitudes towards LTC facilities. Despite feeling uneasy and losing confidence in the system, they recognised their dependence on it [5]. Similarly to older adults and staff, relatives frequently cited understaffing and institutionalisation as major concerns. They observed the constant turnover of caregivers, which meant that many staff were unfamiliar with family values or the personal histories of older people [38]. The shortage of qualified personnel also led to inconsistencies in care quality, with some caregivers lacking formal training, further heightening relatives’ anxieties [6].

Relatives criticised paternalistic care practices, whereby daily routines such as bathing schedules and bedtime were predetermined and non-negotiable. This rigidity not only limited autonomy for older adults and their families but also contributed to a lack of compassion, with staff rarely attending to spiritual or emotional needs [7,38]. Despite their dissatisfaction, many relatives reported surrendering part of their autonomy in exchange for what they perceived as “professional care” [5].

#### 3.3.2. Expectations for an Ethical, Caring Culture

Relatives emphasised that LTC facilities should foster an ethical and caring culture that integrates compassion and individualisation into everyday practice. They argued that ethics should be embedded in both institutional policies and the conduct of individual caregivers, rather than reduced to routinised or purely instrumental tasks [39].

##### Desire for Compassionate, Respectful Interactions

Compassionate care was seen as fundamental to dignity, enabling older adults to feel autonomous, acknowledged, and valued. Relatives highlighted small but meaningful gestures as particularly impactful, such as staff volunteering to do “extra things” [5]. Examples included daily handshakes, patient and kind listening to individuals with dementia, recognising emotions and responding sensitively (e.g., not compelling participation in group activities during low moods), and attending to everyday details such as eating habits, flowers, or tidy surroundings [6,38,39]. Attending to aesthetic needs, such as helping older people to dress well, was also described as a way of enhancing dignity.

Relatives further stressed the importance of “who provides the care”. Compassionate and respectful practices were most often associated with staff who demonstrated genuine concern for older adults and recognised qualities in them that others might overlook. Such attentiveness was said to foster familiarity and trust, forming the basis of a dignified model of care [39].

##### Need for Individualised Autonomy and a Homelike Environment

Relatives frequently highlighted the importance of living arrangements that preserve both autonomy and a sense of continuity with past lives. They expressed a strong preference for single rooms that feel homely, ideally equipped with private bathrooms, personal furnishings, and cherished belongings. Such environments were seen as contributing to dignity and a sense of “rootedness”, as familiar possessions helped transform the “previous home” into the “new home”. In contrast, being forced to share a room was regarded as undermining dignity, since the habits of roommates or unexpected events such as sudden illness or death could cause distress [6].

Individualised autonomy was also emphasised, particularly in relation to decision-making. Relatives recognised that older adults valued the ability to make choices, but they cautioned that excessive decision-making could be exhausting and burdensome. What was considered most essential for preserving dignity was not unlimited autonomy, but the opportunity to participate in significant decisions, especially those concerning health and well-being [6,39]. This view corresponded with the theme of “frailty and substituted decision-making” discussed in the residents’ perspectives, suggesting that shared decision-making models may best balance autonomy with support.

## 4. Discussion

This review systematically identified, summarised, and synthesised qualitative studies on the dignity of older adults in LTC facilities. Among older adults, staff, and relatives, common challenges included understaffing, the pursuit of compassionate care, and the need for adaptability and acceptance. Geographical variation revealed that Nordic countries have more established dignity-oriented LTC systems, while Asian contexts remain underexplored. The proposed triangular model highlights that dignity is upheld through reciprocal relationships among residents, staff, and families. To operationalise this model, practical and policy actions are needed to strengthen the workforce, promote equity and person-centred care, and foster resident and family engagement within LTC institutions.

### 4.1. Shared Challenges and Divergent Perspectives on Dignity

In LTC contexts, human dignity represents intrinsic worth, while social dignity, or dignity of identity, is shaped by relationships and the care environment [15,16,17]. Our themes reveal tensions between these dimensions: institutional routines and dependence threaten autonomy and identity, whereas empathy, attentiveness, and mutual respect help restore dignity. Thus, dignity in LTC is not fixed but continuously negotiated through daily interactions among residents, staff, and relatives.

Across all three groups, understaffing and heavy routine workloads were identified as central barriers to dignified care. Older adults and their relatives frequently described how staff, constrained by time and paperwork, lacked the capacity to provide high-quality, person-centred support. Long waiting times for assistance were common, reinforcing the sense that care was task-driven rather than tailored [30,35]. Staff themselves recognised this tension: although motivated to deliver person-centred care, they acknowledged that administrative demands and excessive routine duties limited their ability to do so. High staff turnover was also consistently reported. Staff attributed this problem to the heavy workload and the low social status of aged-care practitioners, whereas older adults and their families were more concerned about the resulting discontinuity of care, the variability in professionalism, and the difficulty of adaptation when staff changed frequently [34,35,36]. Prior studies have also identified low rates of pay, unsociable working hours, insufficient benefits, and limited career advancement opportunities as key contributors to workforce instability in LTC settings [40,41]. High turnover is particularly prevalent among direct care workers, such as certified nursing assistants, who provide the majority of hands-on care [42].

Another shared perspective was the value of “compassionate care”. All three groups emphasised that attention to detail could foster a reciprocal relationship: older people felt respected and valued, while staff derived meaning and satisfaction from their work [7,38]. This reciprocity highlights the dual benefit of compassionate practices for both caregivers and care recipients. The principle of “treating people as you would like to be treated” was also mentioned by both staff and relatives, though with different emphases. Staff primarily associated it with protecting privacy, such as ensuring confidentiality and maintaining discretion, while relatives were more concerned about avoiding situations that left older people feeling neglected or abandoned [7,36]. However, compassionate care and the aforementioned understaffing seem to be intertwined and contradictory situations. As indicated in other studies, compassionate care in LTC settings faces multiple challenges, including staff stress, training gaps, institutional constraints, and resident complexity [43]. Nurses typically begin their careers with high levels of compassion, but this tends to diminish during training or early professional years. Front-line staff working with older adults, especially those with dementia, often endure long hours in stressful environments, which can undermine their ability to provide compassionate care [44]. Thus, there is an urgent need for policy-level interventions to improve job quality, career pathways, and working conditions in LTC settings.

Meanwhile, important differences emerged in how adaptability and acceptance were expressed. Older adults often experience significant psychological and emotional challenges when transitioning to LTC facilities [45]. Emotional distress, depressed mood, and difficulty in accepting the new residence are common, especially in cases of involuntary or unplanned admissions, which are associated with increased anxiety, confusion, and even higher mortality rates [46]. Included studies in this review showed that older adults often demonstrated resilience by accommodating the loss of past social relationships and life patterns, striving to adjust to institutional life with perseverance [25,26,27,28,30]. Relatives, by contrast, tended to emphasise reluctant acceptance of a system they viewed as inadequate, surrendering part of their decision-making power in exchange for LTC for their loved ones [5,6]. A care plan developed collaboratively by residents, family members, and staff, and prioritizing the emotional needs of older adults, is necessary.

### 4.2. Dignity of Older Adults in LTC Facilities Across Regions

This review revealed marked geographical differences in the distribution of included studies. Europe accounted for the largest proportion of studies, particularly in Nordic countries such as Sweden and Norway, while only three came from Asia, all focusing exclusively on residents’ perspectives. Such disparities may evolve from policy orientation and welfare models, timing of LTC development, cultural traditions, and variation in service models and funding.

Across the Nordic countries, the emphasis on universal welfare and public responsibility has long shaped both policy and research agendas. Nations such as Denmark and Sweden have developed highly integrated LTC systems that prioritise equality, autonomy, and dignity as central values [47]. These countries feature strong municipal governance, stable funding, and professionalised care networks, providing fertile ground for empirical and qualitative inquiry into residents’ lived experiences [48]. In contrast, regions where LTC relies more heavily on family provision or market forces have tended to focus on service expansion and resource allocation, with less attention paid to subjective aspects such as dignity [49].

The historical trajectory of LTC development also plays a major role. Nordic countries began institutionalising LTC as early as the 1960s and 1970s, establishing a mature infrastructure for both service delivery and research. Many continental European nations followed decades later, while numerous East Asian countries remain at the stage of policy experimentation and pilot implementation [50]. This temporal gap partly explains the concentration of dignity-focused research in regions with long-established LTC systems [51].

Cultural values further shape research focus and care practice. One included study conducted in South Korea found that participants referred more frequently to “feeling abandoned by their children” than those in Western contexts [28]. In Confucian societies such as China and South Korea, elder care remains deeply embedded in family structures, where filial piety defines moral responsibility. This strong family orientation often reduces reliance on formal LTC institutions and may consequently limit the academic focus on institutional dignity [49]. In contrast, Western welfare states place greater emphasis on individual autonomy and social rights, fostering a research culture attentive to person-centred and dignity-oriented care [51].

Furthermore, differences in service models and funding mechanisms reinforce these regional contrasts. As summarised in Table 5, Nordic countries maintain universal, publicly funded LTC systems in which the state assumes primary responsibility and family involvement is minimal [47,48,49]. North America presents a more mixed picture: the United States relies predominantly on market mechanisms, while Canada adopts regionally varied, publicly subsidised models [52]. China, by comparison, remains in the early stages of LTC system development. Care provision is still dominated by families, though rapid growth of institutional care and private-sector participation has occurred in recent years. Ongoing national reforms aim to integrate health and social care, strengthen home- and community-based services, and professionalise the care workforce [52,53].

### 4.3. Building Reciprocal Relationships: A Triangular Model of Dignity Among Older Adults, Staff, and Relatives

This review revealed an apparent tension between institutionalisation and person-centred care. At first sight, institutionalisation seems to limit dignity, as it is linked with “long waits”, unmet needs, and rigid routines. Yet closer reading suggests that the real problem is not institutionalisation itself, but persistent understaffing and a lack of resources. When too few staff are responsible for too many routine tasks, there is little time to listen, respond, or build relationships with older adults. If resources allowed tasks to be shared among more staff, caregivers could devote more attention to personal preferences and deliver truly person-centred care. While some improvements can be made by reducing unnecessary paperwork or simplifying routines, the deeper issues of staffing shortages and limited funding must be addressed.

Adaptability also emerged as a central theme. Older adults showed resilience when adjusting to the loss of past routines and relationships. Their experiences were rarely entirely positive or entirely negative. Instead, dignity was sustained through a gradual process of adaptation. Even when they longed for their former lives, older people found ways to accept institutional living—for example, by joining group activities, keeping personal habits, or adopting a more content outlook. Adaptability can therefore be seen as balancing the desire to “return to the past” with the need to “live in the present”.

**Figure 3:**
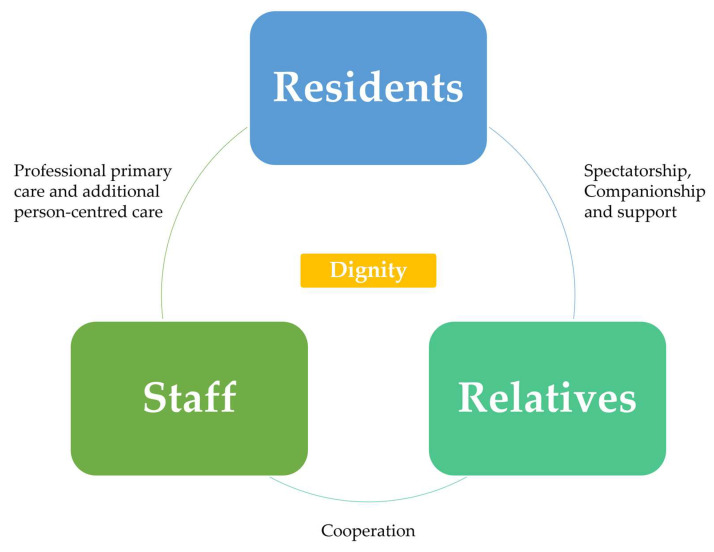
A reciprocal triangular model of dignity in long-term care. The model shows how dignity in long-term care is upheld through reciprocal relationships among older adults, staff, and relatives. Staff provide professional and person-centred care, relatives offer companionship and support, and cooperation between all parties sustains dignity.

Taken together, our findings suggest that dignity in LTC depends on reciprocal relationships among older adults, staff, and relatives (See Figure 3). Older adults are respected for their autonomy and feel safe; staff move beyond routine tasks to engage in meaningful, human interactions; and relatives, as both observers and supporters, gain reassurance when they see trusting bonds between staff and their loved ones. In this triangular model, dignity is sustained by the adaptability of older adults, the professional and person-centred care of staff, and the cooperative involvement of families. Ultimately, dignity is more than a set of care practices. It requires a change in how ageing is understood, and in how staff, relatives, and older adults themselves build respectful and reciprocal relationships in daily life.

### 4.4. Practical and Policy Implications

To effectively implement the proposed triangular model of dignity, policies should address persistent understaffing of LTC facilities, promoting dignity care, and encouraging active engagement of residents and their families.

#### 4.4.1. Optimising the LTC Workforce and Institutional Support

Ensuring a stable and well-trained workforce is the foundation for maintaining dignified care. Improving pay and working conditions is essential for recruitment and retention, as low wages, unsociable hours, and limited benefits remain major drivers of turnover [40,41]. Providing continuing education, supportive supervision, and clear career advancement pathways can improve job satisfaction and professional commitment [54]. Organizational strategies such as adequate staffing ratios, better resource allocation, and regular monitoring of turnover can further enhance job stability and care quality [55]. Identifying and addressing workplace factors that influence satisfaction, such as role clarity, recognition, and participation in decision-making, can also help retain competent caregivers [40].

#### 4.4.2. Promoting Equity in LTC Resources and Dignity Care

Sustaining dignity in LTC requires not only workforce stability but also equitable resource distribution and person-centred service delivery. Countries with mature LTC systems, particularly in Europe, have already integrated dignity care into their welfare models. However, developing systems, such as those in China, still face regional disparities in access. Addressing geographical inequity and optimizing institutional distribution are therefore crucial [52]. Policymakers can coordinate institutional planning, integrate fragmented resources, and encourage collaboration between public and private sectors. Fiscal incentives such as tax reductions may stimulate qualified institutional participation, while increasing overall investment remains essential to ensure equitable access and care quality [56]. After resource optimization, facilities should further implement individualized, dignity-conserving care by offering residents meaningful choices, social participation, and activities that sustain autonomy and well-being [57].

#### 4.4.3. Encouraging Resident and Family Engagement in LTC Life

Successful adaptation to institutional living requires emotional support and meaningful engagement from both staff and families. Relocation often involves loss and identity adjustment, making relational and environmental support vital [58]. Resident-centred and family-inclusive approaches can strengthen residents’ sense of belonging and reduce isolation [59]. LTC settings should be designed to resemble home environments, enhancing privacy, autonomy, and social interaction. Structured exercise and rehabilitation programs can promote mobility, emotional well-being, and independence [57].

### 4.5. Limitations

Although a comprehensive search was conducted across six major English and Chinese databases, studies published in other languages were not captured. This may have led to the omission of relevant qualitative research conducted in regions such as Latin America, Southeast Asia, and Africa. Future reviews incorporating additional language databases or multilingual collaboration would enhance geographical coverage and global applicability. Meanwhile, all team members share a background in medicinal administration, with specialities in clinical medicine, nursing, and statistics. This background may have influenced our interpretation of dignity-related themes toward clinical and institutional aspects. Future reviews that include researchers from fields such as social work or anthropology could offer broader perspectives and further enhance the cross-disciplinary understanding of dignity in LTC.

## 5. Conclusions

This systematic review synthesised qualitative evidence on the dignity of older adults in LTC facilities from the perspectives of residents, staff, and relatives. Across diverse settings, three key insights emerged: first, that dignity is often threatened by understaffing and rigid routines rather than institutionalisation itself; second, that adaptability allows older people to negotiate the tension between longing for the past and living in the present; and third, that dignity is best sustained through reciprocal relationships among older adults, staff, and relatives. Together, these findings inform a triangular model of dignity. Implementing this model requires simultaneous investment in workforce reform, equitable LTC system development, and family-integrated care.

## Figures and Tables

**Table 1:** Previously published reviews on dignity of older adults in LTC settings.

Author	Year	Type of Review	No. of Included Studies	Language(s) of Included Studies	Regions Covered	Perspective(s)
Hasegawa & Ota [19]	2019	Literature review	28	English	Mainly North America and Europe	From older adults
Šaňáková & Čáp [13]	2019	Literature review	14	English	Mainly Europe and North America	From older adults and nurses
Kane and de Vries [20]	2017	Literature review	29	English	Europe, North America, Oceania, and East Asia	From older adults, resident’s family, and staff

**Table 2:** PICo framework for qualitative systematic reviews.

Population (P)	Phenomena of Interest (I)	Context (Co)
Older adults residing in LTC facilities, as well as their relatives and professional caregivers (including nurses and care staff).	Experiences, perceptions, and interpretations of dignity, including factors that preserve or undermine it within LTC settings.	Institutional long-term care environments, such as nursing homes, skilled nursing facilities, and assisted living facilities.

Note: The PICo framework (Population, Phenomena of Interest, and Context) was applied to define the scope of the review and guide the development of the search strategy.

**Table 3:** Search terms used in database searches.

Population	Phenomena of Interest	Context	Methodology
Older people; Old people; Elderly; Aged; Older adult *	Dignity; Personal respect; Respect	Long-term care facility *; Elderly home *; Nursing home *; Nursing facility *; Long-term care setting *; Elderly care facility *; Continuing care retirement communit *	Qualitative study; Qualitative research; Phenomenology; Grounded theory; Interview

Note: Terms within each column were combined using the Boolean operator OR; terms across different columns were combined using AND. Truncation (*) was applied to capture variations in word endings.

**Table 4:** Themes of dignity of older people in LTC facilities.

Perspectives	Themes	Subthemes
1. Residents’ perspectives	1.1. Institutionalisation and loss of autonomy	1.1.1. Rigid routines and staff workload limit autonomy; 1.1.2. “Home” versus “facility”: privacy, freedom, and restriction; 1.1.3. Person-centred care: gap between claim and practice; 1.1.4. Frailty and substituted decision-making.
	1.2. Resilience and adaptation to preserve dignity	1.2.1. Mourning past life and identity changes; 1.2.2. Finding benefits and cherishing small blessings.
2. Staff’s perspectives	2.1. Foundations of dignified care	2.1.1. Professional standards in everyday care; 2.1.2. Partnership with relatives.
	2.2. Implementing person-centred care	2.2.1. Attentiveness to individual details; 2.2.2. Empathetic reciprocity in care.
	2.3. Workforce shortages and workload pressures	2.3.1. Time pressure and paperwork burden; 2.3.2. High turnover and low morale.
3. Relatives’ perspectives	3.1. Unease regarding neglect and indignity	3.1.1. Perceived lack of empathetic care; 3.1.2. Reluctant reliance on the system.
	3.2. Expectations for an ethical, caring culture	3.2.1. Desire for compassionate, respectful interactions; 3.2.2. Need for individualised autonomy and a homelike environment.

**Table 5:** Comparison of LTC Systems: Nordic Countries, North America, and China.

Region	Main Features	Role of State vs. Family	Funding
Nordic Countries	Integrated, comprehensive, universalistic, state-driven, local municipalities responsible	State assumes primary responsibility, low family involvement	Universal coverage, public funding
North America	Mixed models, regional differences, market-driven in USA, combination of state/family in Canada	Shared responsibility between state and family, regional variation	Market-driven (USA), mixed funding (Canada)
China	Early stage of LTC development, infrequent research on dignity, workforce and infrastructure issues	Family plays major role, limited state provision	Developing systems, increasing involvement of private sectors

## Data Availability

No new data were created or analyzed in this study.

## Abbreviations

The following abbreviations are used in this manuscript:

CASP	Critical Appraisal Skills Programme
JBI	Joanna Briggs Institute
LTC	Long-term care
OSF	Open Science Framework
PICo	Population, Phenomena of Interest, and Context
PICO	Population, Intervention, Comparison, Outcome
PRISMA	Preferred Reporting Items for Systematic Reviews and Meta-Analyses
WHO	World Health Organization

## Appendix A

**Table A1:** Sample search strategy.

Database	Search Strategy
PubMed	((older people) [Title/Abstract] OR (old people) [Title/Abstract] OR (elderly) [Title/Abstract] OR (aged)[Title/Abstract] OR (older adult*)) [Title/Abstract] AND ((dignity) [Title/Abstract] OR (personal respect)[Title/Abstract] OR (respect)) [Title/Abstract] AND ((long-term care facilit*) [Title/Abstract] OR (elderly home*) [Title/Abstract] OR (nursing home*)[Title/Abstract] OR (nursing facilit*)[Title/Abstract] OR (assisted living facilit*)[Title/Abstract] OR (long-term care setting*)[Title/Abstract] OR (elderly care facilit*) [Title/Abstract] OR (Continuing care retirement communit*)) [Title/Abstract] AND ((qualitative study) [Title/Abstract] OR (qualitative research) [Title/Abstract] OR (phenomenology) [Title/Abstract] OR (grounded theory) [Title/Abstract] OR (interview)) [Title/Abstract]
Wan Fang (Chinese Electronic Database)	(题名、摘要或关键词:(((老人) or (老年人))) and题名、摘要或关键词:(((尊严) or (尊严权) or (人格尊严) or (人格尊严权)) ) and题名、摘要或关键词:( ((养老院) or (养老机构) or (敬老院) or (老人之家) or (照护)) ) and题名、摘要或关键词:(((定性) or (质性) or (访谈) or (现象学) or (体验) or (个案)))) and Date:2011–2023

1. * (asterisk) indicates an infinite truncation, i.e., any supplementary field. 2. Chinese search terminology: 题名、摘要或关键词 means Title, Abstract or Keywords, 老人/老年人means Elderly, 尊严/尊严权 means Dignity, 人格尊严/人格尊严权 means Human dignity, 养老院/养老机构/敬老院/老人之家 means Nursing home, 照护 means Care, 定性/质性 means Qualitative, 访谈 means Interview, 现象学 means Phenomenology, 体验 means Experience, 个案 means Case.

**Table A2:** Quality appraisal (CASP Qualitative Checklist 2018).

Item	Questions
Section A1: Are the results valid?	1. Was there a clear statement of the aims of the research? 2. Is a qualitative methodology appropriate?
Section A2: Is it worth continuing?	3. Was the research design appropriate to address the aims of the research? 4. Was the recruitment strategy appropriate to the aims of the research? 5. Was the data collected in a way that addressed the research issue? 6. Has the relationship between researcher and participants been adequately considered?
Section B: What are the results?	7. Have ethical issues been taken into consideration? 8. Was the data analysis sufficiently rigorous? 9. Is there a clear statement of findings?
Section C: Will the results help locally?	10. How valuable is the research?

**Table A3:** Critical appraisal results of included studies.

Study	Design	Sampling	Data Collection	Reflexivity	Ethical Issues	Analysis	Findings	Value	Total Score
James, I. et al. [26]	2	2	3	3	3	3	3	3	22
Donnelly, L. et al. [27]	3	3	3	3	3	3	3	3	24
Oosterveld-Vlug, M. G. et al. [30]	3	3	2	2	3	3	3	3	22
Oosterveld-Vlug, M. G. et al. [35]	2	3	3	2	3	3	3	3	22
Heggestad, A. K. et al. [29]	3	3	2	3	3	3	3	3	23
Chang, S. J. [28]	3	2	3	3	3	3	3	3	23
Høy, B. et al. [2]	3	3	3	3	3	3	3	3	24
Bangerter, L. R. et al. [33]	3	3	3	3	3	3	3	3	24
Walker, H. et al. [25]	3	3	2	2	3	3	3	3	22
Caspari, S. et al. [6]	3	2	3	3	3	3	3	3	23
Saeteren, B. et al. [4]	3	2	2	1	3	2	3	3	19
Slettebø, Å et al. [32]	3	2	3	3	3	3	3	3	23
Cai Qian et al. [60]	3	3	3	3	2	3	3	3	23
Wang Chengshuang et al. [31]	3	3	3	1	2	3	3	3	21
Ostaszkiewicz, J. et al. [36]	3	3	3	2	3	3	3	3	23
Blank, M. L. et al. [34]	3	3	3	3	3	3	3	3	24
Oosterveld-Vlug, M. G. et al. [61]	3	3	3	3	3	3	3	3	24
Fetherstonhaugh, D. et al. [37]	3	3	3	3	3	3	3	3	24
Lohne, V. et al. [5]	3	2	3	3	3	3	3	3	23
Mlinar Reljić, N. et al. [38]	3	3	3	3	3	3	3	3	24
Rehnsfeldt, A. et al. [39]	3	1	2	2	3	2	3	3	19
Caspari, S. et al. [3]	3	2	3	3	3	3	3	3	23
Nåden, D. et al. [7]	3	3	2	1	3	3	3	3	21
Hall, S. et al. [62]	3	3	3	3	3	3	3	3	24
Gallagher, A. et al. [63]	3	2	3	2	3	2	3	3	21
Næss, A. et al. [64]	3	3	2	3	3	1	3	3	21
Heggestad, A. K. et al. [65]	3	3	3	3	3	3	3	3	24

**Table A4:** Summary of studies.

Country or Region	Aim	Population	Methodology/ Data Collection/ Data Analysis	Findings	CASP Total Score
Residents’ Perspectives					
Sweden [26]	Residents’ experience and knowledge about a meaningful daily life in NH	25 residents from 5 NHs	Hermeneutic approach/ Participatory appreciative action reflection (PAAR)/ Life-world hermeneutic approach	It made sense to have enough space to be themselves, to feel safe and to encounter some events that break the routine. Reciprocity in the relationship between residents and staff was a determining factor in achieving meaningful lives of residents.	22
Canada [27]	Residents’ perceptions on LTC facilities purporting to offer person-centered care	20 residents from 7 LTC facilities	Framework analysis/ Interpretive inquiry/ Grounded theory	The institutionalization of NHs and the motivation of staff affected the care environment and thus changed the dignity experience of residents. Despite not experiencing sufficient person-centred care, residents struggled to adapt to life in NHs.	24
Netherlands [61]	Changes in nursing home residents’ dignity over time	22 residents in 4 NHs	Qualitative descriptive methods/ Multiple in-depth interview/ Thematic analysis	On the one hand, being in control of their lives enhanced residents’ experience of dignity and satisfaction with NH life. On the other hand, the residents’ ability to adapt, physical condition and social skills determined whether they were regarded as worthwhile people.	22
Netherlands [30]	Residents’ experience of personal dignity	30 recently admitted residents of the general medical wards of 4 NHs	Qualitative descriptive methods/ Interview (Uncertain form)/ Constant comparison approach	Illnesses indirectly influenced the residents’ individual, relational and societal selves. Good coping capabilities, supportive social network and professional care were supportive aspects toward enhancing dignity.	22
Norway [29]	How life in NH may affect experiences of dignity among persons with dementia	15 residents in 2 NHs	Phenomenological-hermeneutic approach/ observation (field notes) and semi-structured interview/ Kvale’s three levels in interpretation	The needs for confirmation, freedom and belonging were intertwined and were linked to a person’s experience of dignity.	24
South Korea [28]	Experience of life among NH residents	11 residents in 2 NHs	Phenomenological approach/ Individual interview/ Colaizzi’s phenomenological method	Nine themes influenced the lived experience of NH residents: Giving up on one self, growing apart from familiar relationships, perceiving the monotony of daily life as suffering, feeling anxious about one’s future upon observing other residents, being dissatisfied with the lack of consideration for individualized care, developing interpersonal skills for communal life, missing the daily routines of their past lives, feeling optimistic about living in a nursing home and having a strategy for the remainder of life in the nursing home.	23
Scandinavia [2]	Residents’ view of maintaining dignity	28 residents in 6 NHs	Qualitative descriptive method/ Individual interview/ Phenomenological and hermeneutic approach	Being involved as a human being, being involved as the person one was and strived to become and being involved as an integrated member of the society were 3 core themes of residents’ perspectives of maintaining dignity.	24
USA [33]	NH residents’ care preferences	337 residents in 35 NHs	Qualitative descriptive method/ Systematic ground up data-collection method/ Content analysis	The work ethic of nursing home staff (i.e., respect for residents), communication, professionalism, and courtesy were considered to be the top four nursing home resident care preferences.	24
Australia [25]	Lived experience and perceptions of older people in residential aged care facilities (RACFs) in Australia	18 residents in 5 RACFs	Phenomenological approach/ Semi-structured interview/ Thematic analysis	Loss of autonomy, dignity, control and important relationships, and resigned acceptance were main aspects contributed to the reality of RACF life.	22
Scandinavia [3]	NH residents’ experience of freedom	28 residents in 6 NHs	Explorative and hermeneutic design/ Semi-structured interview/ Hermeneutic interpretation	Achieving the experience of freedom required a balance between autonomy and paternalism. Residents’ internal and external freedoms were intertwined. Passive dependency under institutionalised care was an extra burden for residents.	23
Scandinavia [4]	What older NH residents do to preserve dignity	28 residents in 6 NHs	Hermeneutic approach/ Interview (Uncertain form)/ Thematic analysis	There was a series of dialectics in maintaining the dignity of NH residents: institutionalization versus home-like, freedom versus restriction, health versus suffering, achievement versus elimination, dignity versus humiliation. Despite this, residents tried to strike a balance and adapt from it.	19
Scandinavia [32]	Residents’ experience of dignity through meaningful and enjoyable activities	28 residents in 6 NHs	Explorative and hermeneutic design/ Semi-structured interview/ Content analysis	Activities were important for residents to experience dignity in their daily life in NHs. However, it was important that activities were tailored to the individual and that residents were able to actively participate.	23
China [60]	NH residents’ understanding of dignity	16 residents in 3 endowment institutions	Qualitative descriptive method/ Semi-structured interview/ Thematic analysis	Inability to control the body, unsatisfactory quality of care and lack of emotional support were 3 main factors that undermined dignity. Security of life, partial autonomy and good caring-culture were 3 main factors that preserved dignity.	24
China [31]	Older people’s lived experience in medical-nursing combined pension institutions	21 residents from 4 medical-nursing combined pension institutions	Qualitative descriptive method/ Semi-structured interview/ Thematic analysis	To improve the lived experience of residents, medical-nursing combined pension institution should focus on basic life care, dignified privacy care and spiritual belonging care.	22
Staff’s Perspectives					
Australia [36]	NH staff’s beliefs and expectations about “quality continence care”	19 staffs in a NH	Qualitative descriptive method/ Semi-structured interview/ Content analysis	Appropriate use of pads, education of staff and relatives on incontinence management, provision of timely incontinence care, consideration of residents’ preferences and communicating sensitively are necessary to protect residents’ dignity.	23
New Zealand [34]	Staff’s understandings of spiritual care and spirituality	19 staffs in 3 LTC facilities	Framework analysis/ Semi-structured interview/ Thematic analysis	Spirituality and spiritual care are different. To provide spiritual care, 5 steps need to be accomplished: information gathering, facilitation, companionship, end-of-life care and personal counseling.	24
Netherlands [35]	Staff’s experiences with preserving dignity within NH	13 physicians and 15 nurses	Qualitative descriptive method/ Semi-structured interview/ Thematic analysis	In staff’s view, keeping residents’ individuality, treat residents as staff would like to be treated, provide general dignity-conserving care and properly handling conflicting values in promoting dignity are essential for maintain residents’ dignity.	24
Australia [37]	How staff in RACFs perceive that they support decision-making for people with dementia	80 direct care staff members in 14 RACFs	Grounded theory methodology/ Semi-structured interview and focus group interview/ Grounded theory	When providing care to a resident with dementia, caregivers should simplify the process of making decisions, know the resident and negotiate a compromise, if necessary.	24
Relatives’ Perspectives					
Scandinavia [5]	Family caregivers’ experiences of dignity of residents in NHs	29 family caregivers in 6 NHs	Phenomenological-hermeneutic approach/ Semi-structured interview/ Hermeneutic interpretation	Relatives empathise with residents’ experiences of dignity in nursing homes and are upset after seeing and experiencing incidents of indignity, yet they have to rely on the nursing home care system.	23
Slovenia [38]	Family members experiences with the spiritual care for their family members living with dementia in NHs	12 relatives in one NH	Phenomenological-hermeneutic approach/ In-depth face-to-face individual interviews/ Phenomenological hermeneutical analysis	The institutionalization and lack of spiritual care in nursing homes is distressing for relatives of people with dementia. Relatives expect respectful and compassionate care.	24
Scandinavia [39]	Individual variations in caring cultures in relation to dignity	28 family caregivers in 6 NHs	Hermeneutic epistemology/ In-depth interview (Uncertain form)/ Hermeneutic interpretation	Relatives expect an ethical contexts and caring acts in a caring culture that is characterised by caring communion, hospitality, ‘at-home-ness’, person-centredness and ‘the little extra’.	19
Scandinavia [6]	Relatives’ experience of existential needs and concerns in NHs	28 relatives of residents in 6 NHs	Hermeneutic approach/ Semi-structured interviews/ Kvale’s three levels in interpretation	The dignity and existential needs of residents relate to five aspects: ‘comfortable, homely and practical room’, ‘close contact with family, friends and with the staff’, ‘aesthetic needs and concerns’, ‘ethical needs and intrinsic values’ and ‘cultural and spiritual needs and concerns’.	23
Scandinavia [7]	How is residents’ dignity maintained, promoted or deprived from the perspective of family caregivers	28 relatives of residents at 6 NHs in Scandinavia	Explorative and hermeneutic design/ Individual research interview/ Hermeneutic interpretation	Relatives describe the residents’ experience of dignity in a nursing home as a feeling of being abandoned, which involves 6 themes: deprived of the feeling of belonging, deprived of dignity due to acts of omission, deprived of confirmation, deprived of dignity due to physical or psychological humiliation and deprived of parts of life.	21
Multiple Perspectives					
UK [62]	Explore and compare the views of care providers, residents and their families on dignity and how to maintain it	33 care home managers, 29 care assistants, 18 care home nurses, 10 community nurses, 16 residents and 15 members of residents’ families	Qualitative descriptive method/ Semi-structured interview/ Framework approach	The most prevalent themes of maintaining dignity for residents of care homes are: “independence” and “privacy”, followed by “comfort and care,” “individuality,” “respect,” “communication,” “physical appearance” and “being seen as human.”	24
UK [63]	How best to translate the concept of dignity into care home practice	4 Action Research Groups (ARG) with 6-9 volunteers RNs or care workers, and 4 Residents and Relatives Groups (RRG) with 1-5 residents plus 1-4 relatives per group in 4 care homes	Action research/ Group discussion and individual interview/ Thematic analysis	Hard copy and online versions of a dignity toolkit, with tailored versions for participating care homes, were developed.	21
Norway [64]	How care workers make efforts to recognize residents’ selves	Observation: 5 NHs Interviews: 48 employees	Ethnographic fieldwork/ Observation (field notes) and semi-structured interviews/ Thematic analysis	Assisted self-presentation helps residents to express themselves and is implemented in the following steps: shaping social situations, preparing for social performance, prompting of personal narratives and downplaying inappropriate behaviour.	21
Norway [65]	What patients and their relatives experience as promoting and threatening to dignity	7 relatives of patients with dementia in 2 NHs	Phenomenological-hermeneutic approach/ Observation (field notes) and semi-structured interview/ Kvale’s three levels in interpretation	Confirming, person-centred and relational care involving time and resources in the care relationship will promote the dignity of residents, while task-centred care will threaten the dignity of residents.	24

**Table A5:** PRISMA 2020 Checklist.

Section and Topic	Item #	Checklist Item	Location Where Item is Reported
TITLE			
Title	1	Identify the report as a systematic review.	P1 (Title Page)
ABSTRACT			
Abstract	2	See the PRISMA 2020 for Abstracts checklist.	P1 (Structured abstract)
INTRODUCTION			
Rationale	3	Describe the rationale for the review in the context of existing knowledge.	P1-2 (Introduction)
Objectives	4	Provide an explicit statement of the objective(s) or question(s) the review addresses.	P3 (PICo framework)
METHODS			
Eligibility criteria	5	Specify the inclusion and exclusion criteria for the review and how studies were grouped for the syntheses.	P3 (2.2. Eligibility Criteria)
Information sources	6	Specify all databases, registers, websites, organisations, reference lists and other sources searched or consulted to identify studies. Specify the date when each source was last searched or consulted.	P4 (2.3. Search Strategies)
Search strategy	7	Present the full search strategies for all databases, registers and websites, including any filters and limits used.	P4 (2.3. Search Strategies)
Selection process	8	Specify the methods used to decide whether a study met the inclusion criteria of the review, including how many reviewers screened each record and each report retrieved, whether they worked independently, and if applicable, details of automation tools used in the process.	P4 (2.4. Study Selection and Quality Appraisal and 2.5. Data Extraction and Synthesis)
Data collection process	9	Specify the methods used to collect data from reports, including how many reviewers collected data from each report, whether they worked independently, any processes for obtaining or confirming data from study investigators, and if applicable, details of automation tools used in the process.	P4 (2.4. Study Selection and Quality Appraisal and 2.5. Data Extraction and Synthesis)
Data items	10a	List and define all outcomes for which data were sought. Specify whether all results that were compatible with each outcome domain in each study were sought (e.g., for all measures, time points, analyses), and if not, the methods used to decide which results to collect.	P4 (2.4. Study Selection and Quality Appraisal and 2.5. Data Extraction and Synthesis)
	10b	List and define all other variables for which data were sought (e.g., participant and intervention characteristics, funding sources). Describe any assumptions made about any missing or unclear information.	P4 (2.4. Study Selection and Quality Appraisal and 2.5. Data Extraction and Synthesis)
Study risk of bias assessment	11	Specify the methods used to assess risk of bias in the included studies, including details of the tool(s) used, how many reviewers assessed each study and whether they worked independently, and if applicable, details of automation tools used in the process.	P4 (2.4. Study Selection and Quality Appraisal and 2.5. Data Extraction and Synthesis)
Effect measures	12	Specify for each outcome the effect measure(s) (e.g., risk ratio, mean difference) used in the synthesis or presentation of results.	P4 (2.5. Data Extraction and Synthesis)
Synthesis methods	13a	Describe the processes used to decide which studies were eligible for each synthesis (e.g., tabulating the study intervention characteristics and comparing against the planned groups for each synthesis (item #5)).	P4 (2.5. Data Extraction and Synthesis)
	13b	Describe any methods required to prepare the data for presentation or synthesis, such as handling of missing summary statistics, or data conversions.	P4 (2.5. Data Extraction and Synthesis)
	13c	Describe any methods used to tabulate or visually display results of individual studies and syntheses.	P4 (2.5. Data Extraction and Synthesis), and Appendix A Table A3
	13d	Describe any methods used to synthesize results and provide a rationale for the choice(s). If meta-analysis was performed, describe the model(s), method(s) to identify the presence and extent of statistical heterogeneity, and software package(s) used.	P4 (2.5. Data Extraction and Synthesis)
	13e	Describe any methods used to explore possible causes of heterogeneity among study results (e.g., subgroup analysis, meta-regression).	Not applicable (qualitative synthesis only)
	13f	Describe any sensitivity analyses conducted to assess robustness of the synthesized results.	Not applicable (qualitative synthesis only)
Reporting bias assessment	14	Describe any methods used to assess risk of bias due to missing results in a synthesis (arising from reporting biases).	P4 (2.4. Study Selection and Quality Appraisal) and Appendix A Table A4
Certainty assessment	15	Describe any methods used to assess certainty (or confidence) in the body of evidence for an outcome.	Not applicable (qualitative synthesis only)
RESULTS			
Study selection	16a	Describe the results of the search and selection process, from the number of records identified in the search to the number of studies included in the review, ideally using a flow diagram.	P5 (Results and Figure 1)
	16b	Cite studies that might appear to meet the inclusion criteria, but which were excluded, and explain why they were excluded.	P5 (Results and Figure 1)
Study characteristics	17	Cite each included study and present its characteristics.	Appendix A Table A4
Risk of bias in studies	18	Present assessments of risk of bias for each included study.	Appendix A Table A3 (CASP scores)
Results of individual studies	19	For all outcomes, present, for each study: (a) summary statistics for each group (where appropriate) and (b) an effect estimate and its precision (e.g., confidence/credible interval), ideally using structured tables or plots.	Appendix A Table A4 (summaries per study)
Results of syntheses	20a	For each synthesis, briefly summarise the characteristics and risk of bias among contributing studies.	P4-11 (Results, Table 3, and Table 4)
	20b	Present results of all statistical syntheses conducted. If meta-analysis was done, present for each the summary estimate and its precision (e.g., confidence/credible interval) and measures of statistical heterogeneity. If comparing groups, describe the direction of the effect.	Not applicable (qualitative synthesis only)
	20c	Present results of all investigations of possible causes of heterogeneity among study results.	Not applicable (qualitative synthesis only)
	20d	Present results of all sensitivity analyses conducted to assess the robustness of the synthesized results.	Not applicable (qualitative synthesis only)
Reporting biases	21	Present assessments of risk of bias due to missing results (arising from reporting biases) for each synthesis assessed.	P4 (Results) and Appendix A Table A3 (CASP scores)
Certainty of evidence	22	Present assessments of certainty (or confidence) in the body of evidence for each outcome assessed.	Not applicable (qualitative synthesis only)
DISCUSSION			
Discussion	23a	Provide a general interpretation of the results in the context of other evidence.	P11
	23b	Discuss any limitations of the evidence included in the review.	P15-16 (4.5. Limitations)
	23c	Discuss any limitations of the review processes used.	P15-16 (4.5. Limitations)
	23d	Discuss implications of the results for practice, policy, and future research.	P15 (4.4. Practical and policy implications)
OTHER INFORMATION			
Registration and protocol	24a	Provide registration information for the review, including register name and registration number, or state that the review was not registered.	P3 (2. Materials and Methods)
	24b	Indicate where the review protocol can be accessed, or state that a protocol was not prepared.	Not applicable. This is a retrospective registration (“registration following analysis of the data”), and the full manuscript has been attached to the registration record.
	24c	Describe and explain any amendments to information provided at registration or in the protocol.	Not applicable. We made no amendments to the information provided at registration.
Support	25	Describe sources of financial or non-financial support for the review, and the role of the funders or sponsors in the review.	P16 (Funding)
Competing interests	26	Declare any competing interests of review authors.	P16 (Conflicts of Interest)
Availability of data, code and other materials	27	Report which of the following are publicly available and where they can be found: template data collection forms; data extracted from included studies; data used for all analyses; analytic code; any other materials used in the review.	P16 (Data Availability Statement)
